# A Single Whole-Body Low Dose X-Irradiation Does Not Affect L1, B1 and IAP Repeat Element DNA Methylation Longitudinally

**DOI:** 10.1371/journal.pone.0093016

**Published:** 2014-03-27

**Authors:** Michelle R. Newman, Pamela J. Sykes, Benjamin J. Blyth, Eva Bezak, Mark D. Lawrence, Katherine L. Morel, Rebecca J. Ormsby

**Affiliations:** 1 Flinders Centre for Innovation in Cancer, Flinders University and Medical Centre, Bedford Park, South Australia, Australia; 2 Department of Medical Physics, Royal Adelaide Hospital, Adelaide, South Australia, Australia; Victor Chang Cardiac Research Institute, Australia

## Abstract

The low dose radioadaptive response has been shown to be protective against high doses of radiation as well as aging-induced genomic instability. We hypothesised that a single whole-body exposure of low dose radiation would induce a radioadaptive response thereby reducing or abrogating aging-related changes in repeat element DNA methylation in mice. Following sham or 10 mGy X-irradiation, serial peripheral blood sampling was performed and differences in Long Interspersed Nucleic Element 1 (L1), B1 and Intracisternal-A-Particle (IAP) repeat element methylation between samples were assessed using high resolution melt analysis of PCR amplicons. By 420 days post-irradiation, neither radiation- or aging-related changes in the methylation of peripheral blood, spleen or liver L1, B1 and IAP elements were observed. Analysis of the spleen and liver tissues of cohorts of untreated aging mice showed that the 17–19 month age group exhibited higher repeat element methylation than younger or older mice, with no overall decline in methylation detected with age. This is the first temporal analysis of the effect of low dose radiation on repeat element methylation in mouse peripheral blood and the first to examine the long term effect of this dose on repeat element methylation in a radiosensitive tissue (spleen) and a tissue fundamental to the aging process (liver). Our data indicate that the methylation of murine DNA repeat elements can fluctuate with age, but unlike human studies, do not demonstrate an overall aging-related decline. Furthermore, our results indicate that a low dose of ionising radiation does not induce detectable changes to murine repeat element DNA methylation in the tissues and at the time-points examined in this study. This radiation dose is relevant to human diagnostic radiation exposures and suggests that a dose of 10 mGy X-rays, unlike high dose radiation, does not cause significant short or long term changes to repeat element or global DNA methylation.

## Introduction

Age-associated changes that contribute to an overall decline in genomic stability are well documented. These include the accumulation of unrepaired DNA lesions, reduced levels of proteins involved in maintaining genomic integrity and changes in telomere length [1]–[4]. It has also been suggested that a loss of global DNA methylation [5], [6] may contribute to genomic instability since DNA methylation plays a role in preventing the expression of oncogenes and transposable DNA repeat elements, which can disrupt gene expression through transposition and integration into other sites across the genome. Although studies indicate that DNA methylation patterns and repeat element transposition drive genetic diversity [7], [8], the increased frequency of transposition events in aging cells can lead to increased genomic instability (reviewed in [9]). Supporting this is the evidence that in aging mice, an increase in transcription and subsequent transposition of transposable elements such as LINE1 (L1), B1 and the Intracisternal-A-Particle (IAP) has been correlated with a reduction in (global or repeat element) DNA methylation [10]–[12]. Human studies have shown that in older individuals (>60 years of age) there is a loss of repeat element DNA methylation [6], [13]–[15], and a strong correlation between L1 hypomethylation and cancers such as colon and lung cancer [16]–[22]. The DNA damaging effects of high doses of ionising radiation are greater when DNA methylation levels are reduced, resulting in greater radiation-induced genomic instability [23]–[29]. In contrast, low doses of radiation (<100 mGy) have been shown to reduce the level of endogenous DNA damage such as micronuclei that accumulates with age [30], suppress the development of cancers [31]–[36] and increase lifespan [33], [37]._ENREF_30 This is termed a low-dose radioadaptive response. Based on these observations, we sought to determine if the radioadaptive response (a single whole-body 10 mGy X-irradiation) could reduce or prevent the predicted decline in peripheral blood (PB) repeat element (L1, B1 and IAP) DNA methylation with aging, utilising serial blood sampling. Repeat element methylation was assessed by high resolution melt analysis [38]. This is the first report of a longitudinal study of murine PB L1, B1 and IAP repeat element methylation changes following exposure to a single low dose X-irradiation, at a dose (10 mGy) relevant to human diagnostic medical X-ray exposures.

## Materials and Methods

### Mice

All experiments involving the use of animals were approved by the Flinders University Animal Welfare Committee and the South Australian Pathology/Central Health Network Animal Ethics Committee (approval numbers: 736-09 and 11–10 respectively). C57Bl/6 mice were maintained in micro-isolators, on a 12 hour light/dark cycle and were quality controlled for viruses, parasites and bacteria, and with standard chow and water provided *ad libitum*. Mice described as untreated did not undergo any of the experimental procedures, removal from the Flinders University School of Medicine Animal Facility, blood sampling or sham-irradiation. Ages and numbers of mice used are outlined in Table 1.

**Table 1:** The age (months) and numbers of male and female mice at peripheral blood sampling three days prior to irradiation and at the final time-point from Study 1 (pilot study) and Study 2.

			*Dose (mGy)*					
			0			10		
	Time post-irradiation (d)		*n*	Age (months)	*SD*	*n*	Age (months)	*SD*
Study 1 (pilot study)#	−3	Male	5	4.36	*0.41*	5	3.95	*0.75*
		Female	5	4.05	*0.34*	5	4.4	*0.46*
	299	Male	2	14	*0.6*	3	14.09	*0.97*
		Female	5	13.98	*0.34*	5	14.32	*0.46*
Study 2#	−3	Male	10	4.94	*0.89*	10	4.66	*1.05*
		Female	10	4.27	*0.87*	10	4.19	*0.72*
	420	Male	8	18.95	*0.86*	6	18.61	*1.05*
		Female	9	18.11	*0.9*	9	18.07	*0.77*

# Note: only mice alive at the end of the studies were analysed at all time-points.

### Irradiation

Mice aged 4–6 months were transported to the Department of Medical Physics (Royal Adelaide Hospital, Adelaide, South Australia), where they were restrained in a Perspex box (6 mm thick Perspex) with multiple ventilation holes, which was placed upright against a block of solid water 8 cm thick to provide full backscatter. Mice were whole-body irradiated with 10 mGy using an attenuated 140 kVp X-ray beam (8 mm Al half value layer) from a Gulmay D3150 superficial X-ray unit as previously described [39]. A 2 mm thick Cu attenuator was attached to the collimator to reduce the dose-rate to 13.9 mGy/min. Mice were irradiated for a total of 0.72 min. After 0.36 min, the Perspex box was rotated by 180° (equivalent to irradiation with two parallel-opposed beams) to ensure uniform dose distribution. All dosimetry calculations were performed by a Medical Physicist (E. Bezak) and dose output calibration of the superficial X-ray unit was performed according to the Institute of Physics and Engineering in Medicine and Biology (IPEMB) protocol [40], [41]. The actual dose-rate after applying distance and filter modifications was verified with a calibrated survey meter (Victoreen). Sham-irradiated control mice were placed in the Perspex box in front of the X-ray machine, but the machine was not turned on. Treated mice were then returned to the Flinders University School of Medicine Animal Facility for the remainder of the experiment.

### Peripheral blood sampling and tissue collection

PB sampling was performed via tail vein puncture. Prior to sampling, cages were placed in front of a heat lamp for several minutes, following which mice were then placed in a holding restraint. The tails were swabbed with ethanol and a small incision was made to the lateral tail vein using a GoldenRod lancet (MEDIpoint Inc). No more than 100 μL of PB was collected in EDTA-collection tubes (Becton-Dickinson) and stored at −20°C until DNA extraction. Pressure was applied to the wound until bleeding ceased prior to returning animals to their cages. At the final time-point, mice were euthanised by CO_2_ asphyxiation following blood sampling. Spleen and liver were isolated and embedded in cryoprotectant (OCT compound, Tissue-Tek), frozen on dry ice and stored at −80°C until subsequent DNA extraction.

### Genomic DNA extraction and bisulphite modification

Genomic DNA was extracted from PB, or for spleen and liver, from 2×5 μm fresh frozen tissue sections cut using a cryostat (Reichert-Jung Cryocut 1800). Genomic DNA was extracted from tissues using the DNeasy DNA Mini extraction kit (Qiagen) as per manufacturer's instructions except that DNA was eluted with 2×100 μL aliquots of Buffer AE. For PB samples, DNA was eluted in 1×100 μL aliquot of Buffer AE. DNA concentration was quantified using a Nanodrop 8000 (Thermo Scientific). Genomic DNA (40 ng for PB, 200 ng for spleen and liver) was bisulphite modified using the Zymo Research EZ DNA Methylation-Gold kit (Zymo Research) as per manufacturer's instructions. Modified DNA was diluted with water (Qiagen) to 10 ng/μL (theoretical amount based on genomic DNA concentration input into the bisulphite modification reaction) and stored at −20°C.

### PCR and HRM analysis

The relative methylation levels of L1, B1 and IAP elements were analysed by high resolution melt (HRM) using PCR primers (Table S1) and conditions which have previously been validated and described in Newman *et al* [38]. PCR amplification using 1 x EpiTect HRM PCR master mix (Qiagen) and 0.75 μM forward and reverse primers (Table S1) was performed on a Rotor-Gene Q (Qiagen) with the following conditions: 95°C for 5 min followed by 35 cycles of 95°C for 20 sec, 30 sec at the appropriate annealing temperature (Table S1) and 72°C for 20 sec. HRM analysis occurred from 73°C–84°C for L1 elements and 65°C–80°C for B1 and IAP elements, rising by 0.1°C/2 sec. Depending on GC content following bisulphite conversion, samples exhibited higher melting temperatures if more methylated, and hence contained more unconverted cytosine, and lower melting temperatures if less methylated. Differences in DNA methylation between samples were quantified using the Net Temperature Shift (NTS), whereby the HRM curve fluorescence values of a sample were subtracted from a nominated methylated control sample, indicating the “shift” of the sample from the control sample. The fluorescence value at each temperature point (0.1°C intervals) within the entire melt range was summed and divided by 100. Thus, the NTS value is an indicator of differences in methylation between samples at the repeat elements, where the greater the shift from the methylated control, the less methylated the sample is.

### Pyrosequencing

Pyrosequencing reactions were performed by EpigenDx (Massachusetts, USA) on a Qiagen-Pyrosequencing PSQ-MD. B1 element HRM-PCR replicates for each sample (amplified with a biotin-tagged forward primer) were combined and aliquoted into 96-well plates in duplicate (as pyrosequencing replicates) in a volume of 10 μL. The target region for analysis (CpGs 1–7) and the pyrosequencing (reverse) primer for B1 elements (5′- CTT TAT AAA CCA AAC TAA CCT C - 3′) were determined using the PSQ Assay (Qiagen).

### Liquid Chromatography-Mass Spectrometry (LC-MS)

Two hundred nanograms of genomic DNA was hydrolysed and assessed for total 5 mdC content as described in Newman *et al* [38]. Relative methylation was calculated based on the ratio of 5 mdC to dG [5 mdC/dG] and normalised to the 5 mdC/dG ratio of a methylated control DNA sample (Zymo Research).

### Statistical analyses

**Figure 1:**
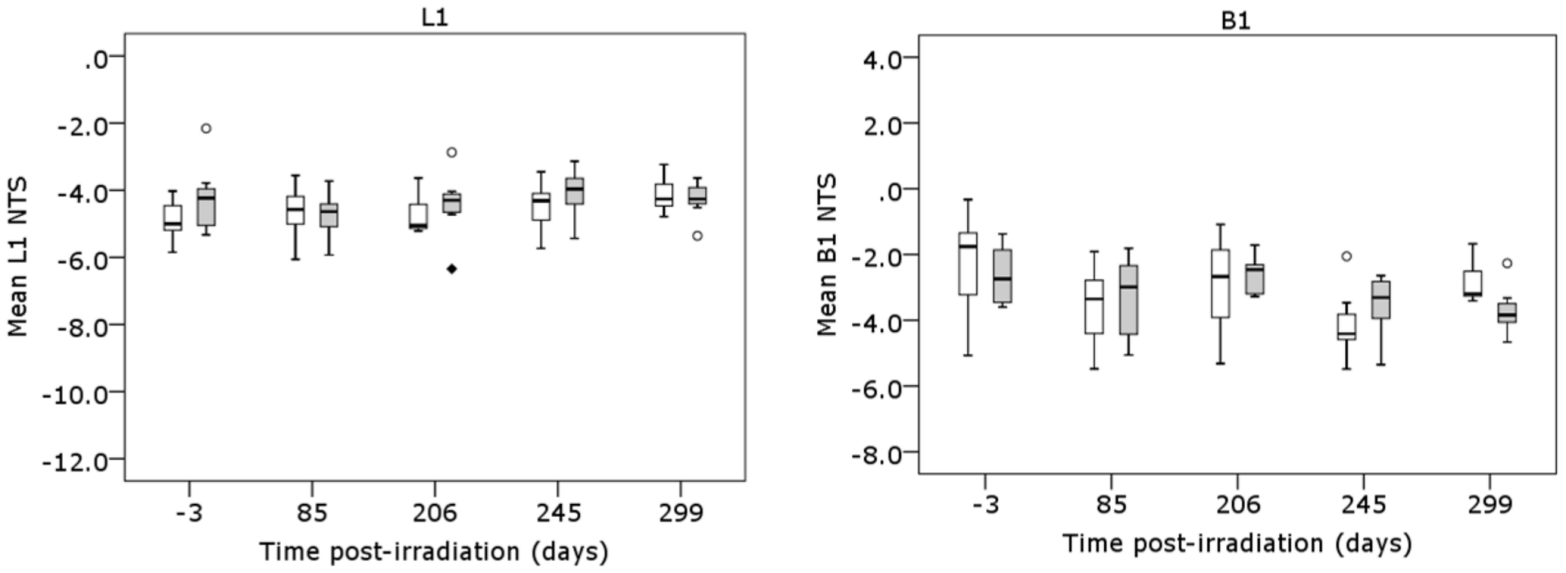
Analysis of the methylation of peripheral blood L1 and B1 repeat elements in aging 10 The Net Temperature Shift (NTS) of L1 and B1 repeat elements from peripheral blood (PB) DNA isolated from C57Bl/6 mice at −3, 85, 206, 245 and 299 days following irradiation with sham (white box; *n* = 7) or 10 mGy (grey box; *n* = 8) X-rays was assessed by high resolution melt analysis following genomic DNA extraction, bisulphite modification and amplification with L1 and B1 primers.

**Figure 2:**
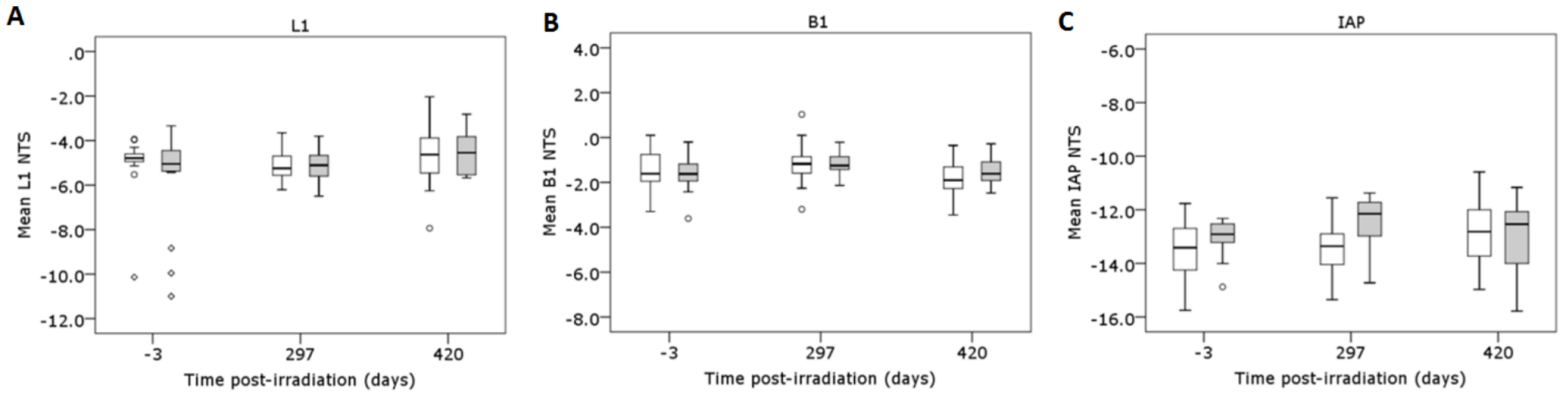
Analysis of the methylation of peripheral blood repeat elements in aging mice 420 days following irradiation with 10-rays. The Net Temperature Shift (NTS) of L1, B1 and IAP repeat elements from PB DNA isolated from C57Bl/6 mice at −3, 297 and 420 days post-irradiation with sham (white box; *n* = 17) or 10 mGy X-rays (grey box; *n* = 15) was assessed by high resolution melt analysis following genomic DNA extraction, bisulphite modification and amplification with L1, B1 and IAP primers.

**Figure 3:**
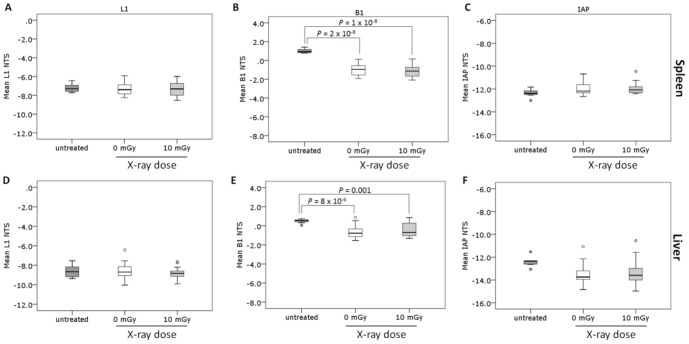
Differences in L1, B1 and IAP repeat element methylation levels between age-matched untreated mice and mice aged 17–19 months at 420 days following irradiation with sham and 10 mGy X-rays. The NTS of L1, B1 and IAP repeat elements in the spleen (A–C) and liver (D–F) tissues of the 17–19 month old mice remaining 420 days following irradiation with sham (white; *n* = 17) or 10 mGy (light grey; *n* = 15) X-rays were compared to a cohort of untreated 17–19 month old mice (dark grey; *n* = 7). The NTS was assessed by high resolution melt following DNA extraction, bisulphite modification and amplification of repeat elements.

**Figure 4:**
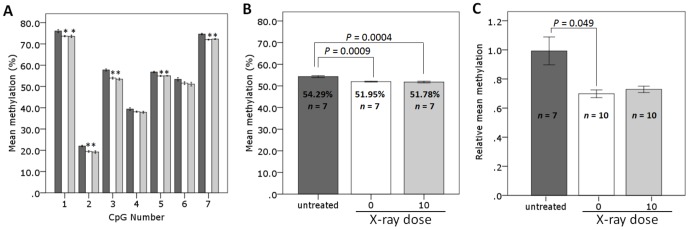
Pyrosequencing and LC-MS analysis of the liver B1 elements of age-matched untreated mice and mice aged 17–19 months at 420 days following irradiation with sham and 10 mGy X-rays. B1-HRM PCR products from liver tissues of 17–19 month old untreated mice (dark grey) and mice at 420 days post-irradiation with sham (white) or 10 mGy (light grey) X-rays were analysed by pyrosequencing (A–B). Liver global methylation levels of 17–19 month old untreated mice (dark grey) and mice at 420 days post-irradiation with sham (white) or 10 mGy (light grey) X-rays were analysed by LC-MS (C). Error bars represent ±1 standard error. *P<0.05 compared to corresponding untreated group.

**Figure 5:**
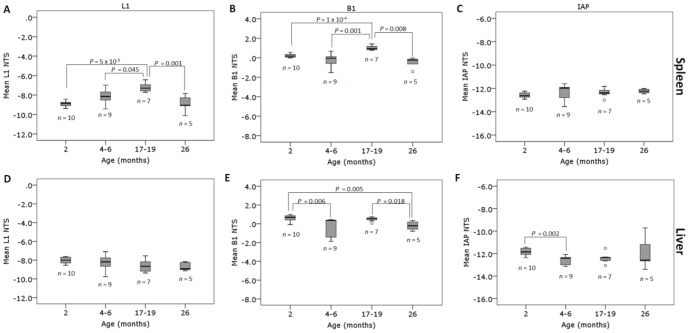
L1, B1 and IAP repeat element DNA methylation in untreated aging mice. The L1, B1 and IAP repeat element methylation in the spleen (A–C) and liver (D–F) tissues of untreated 2 (*n* = 10), 4–6 (*n* = 9), 17–19 (*n* = 7) and 26 (*n* = 5) month old mice was assessed by high resolution melt following DNA extraction, bisulphite modification and amplification.

**Figure 6:**
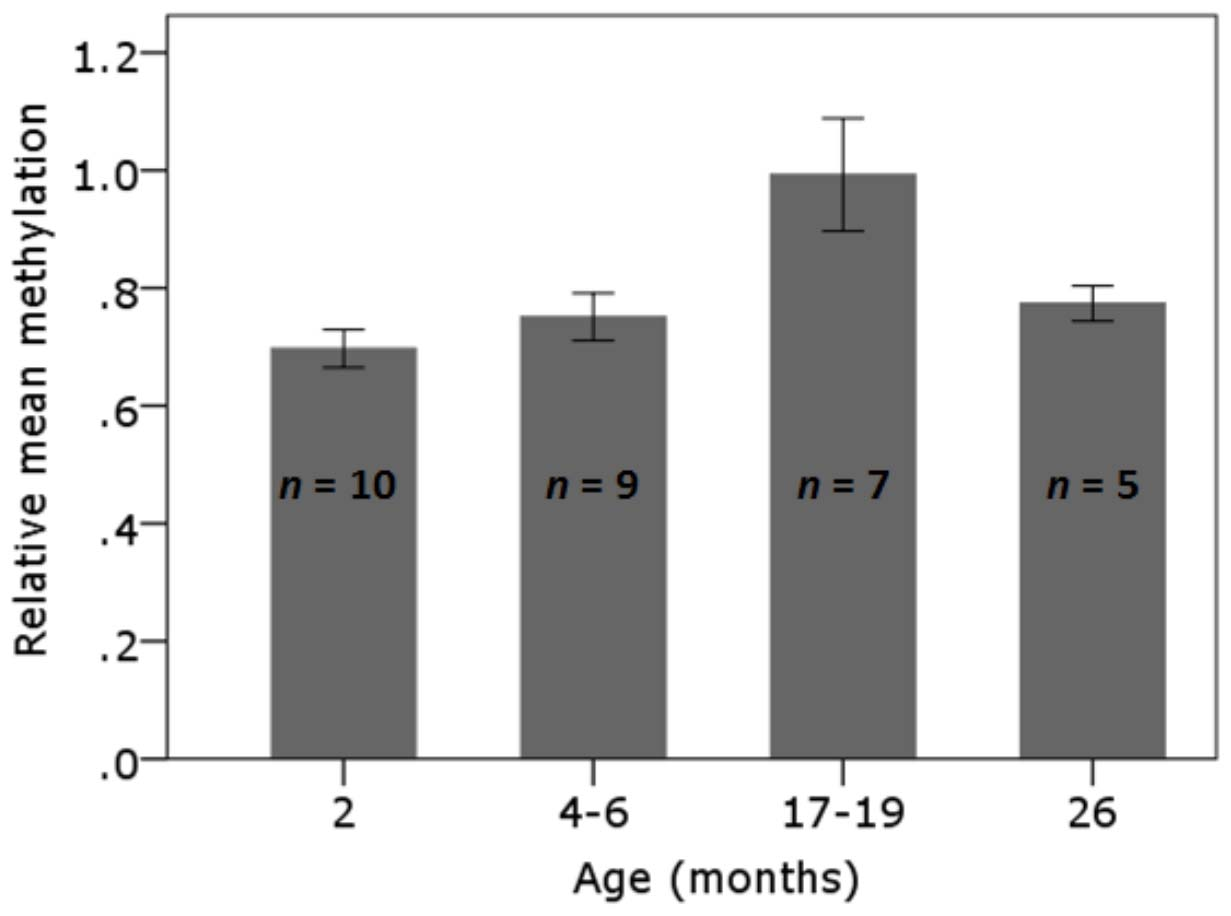
LC-MS analysis of total genomic 5mdC content of liver tissues from untreated mice of different age groups. The relative 5mdC levels of liver tissues from untreated 2 (*n* = 10), 4–6 (*n* = 9), 17–19 (*n* = 7) and 26 (*n* = 5) month old mice were analysed by LC-MS. Error bars represent ±1 standard error.

Data were analysed using the statistical program IBM SPSS Statistics (version 19, IBM Corp.). Data were assessed for normality of distribution using the Shapiro-Wilk test and for homogeneity of variance using the Levene's statistic. Analyses of PB DNA methylation changes over time (Fig 1, Fig 2) were analysed using an ANOVA with repeated measures [42]._ENREF_66 NTS, pyrosequencing and LC-MS data, where three or more groups were present, were analysed using a univariate ANOVA and Bonferroni *post hoc* test where data was parametric and homoscedastic. Where data displayed heteroscedasticity (Fig 3E, Fig 4C, Fig 5B, 5C, 5F and Fig 6), a Welch ANOVA with Games-Howell *post hoc* test was performed. Data determined to be non-parametric (Fig 3C, Fig 5E), were analysed using the non-parametric independent samples Kruskal-Wallis test with significant differences between groups identified using a Mann-Whitney U test with Bonferroni correction. In all cases a *P* value of <0.05 was considered significant. Box plots represent the 25^th^, 50^th^ and 75^th^ percentiles. Box plot error bars indicate the minimum and maximum sample values. Outliers 1.5 times and 3 times the interquartile range are represented as circles and diamonds, respectively. Pyrosequencing and LC-MS error bars indicate ±1 standard error of the mean.

## Results

### Study rationale

Mouse models have provided much of the information that is known about the low dose radioadaptive response. The low dose radioadaptive response is a well-documented phenomenon whereby low doses of radiation can induce a protective effect against DNA damage induced by high doses of radiation and genotoxic chemicals (reviewed in [43]–[45]). While high dose radiation exposure (>0.5 Gy) has been reported to induce changes to DNA methylation, including that of repeat elements, there is no data indicating that this occurs following low dose exposure. A low dose exposure has also been shown to reduce aging-related increases in micronuclei frequency [30]. Aging has been shown, in humans, to correlate with a decline in DNA methylation and an increase in genomic instability [13]–[16], [46]–[48]. Thus, we sought to determine if a low dose of X-irradiation could induce a radioadaptive response in mice thereby reducing or preventing the aging–related decline in DNA methylation. In order to detect changes in methylation over time, peripheral blood was examined as this has been examined extensively in human methylation studies [10], [49]–[52] and the ability to sample this tissue serially enables the analysis of temporal responses to aging and radiation exposure. Many human studies monitor methylation changes over time by analysing the methylation levels of repeat elements such as L1, Alu and Long Terminal Repeat (LTR) elements. This is predominantly due the widespread distribution of these elements, and the evidence that increased transposition activity of these elements due to a loss of methylation can increase the overall genomic instability associated with age [13]–[16], [50]–[52]. There is also evidence that methylation of the murine homologues, L1, B1 (Alu) and IAP (LTR), can display aberrant methylation in aging mice [10], [12], [53]. Based on both the human and mouse studies, we chose to utilise the L1, B1 (Alu homologue) and IAP (murine LTR) elements to monitor age or X-ray induced changes to methylation. Spleen and liver tissues were chosen for analysis at the end of the study as spleen is considered a radiosensitive organ and has been the subject of many radiation and radioadaptive studies [53]–[56] while liver has been investigated in a number of murine aging studies and has been shown to exhibit methylation changes with age [54], [55], [56]. Furthermore, both tissues have been shown to display reduced methylation following high dose radiation exposure [57], [58]. A cohort of age-matched untreated mice was included as a control to account for any potential impact of the sham irradiation procedure on DNA methylation changes. We have used high resolution melt analysis to determine repeat element methylation levels as we have previously demonstrated this to be a high throughput, sensitive and reproducible method for detecting small changes in methylation levels based on differences in melting temperature compared to a methylated control sample [38].

### Analysis of the methylation of DNA repeat elements in aging mice following 10 mGy X-irradiation using repeated blood sampling

In a pilot study, mice (*n* = 10 per treatment group) were sham or 10 mGy X-irradiated following which PB samples were taken at 85, 206, 245 and 299 days post-irradiation (Table 1, Study 1). No significant temporal changes in methylation of the L1 and B1 repeat elements were detected compared to pre-irradiation (-3 days) measurements (*P*>0.05, ANOVA with repeated measures) (Figure 1). A second study was performed with increased sample size (*n* = 20 per treatment group) to account for the greater time post-irradiation (420 days) and associated expected attrition (Table 1, Study 2). Based on the data obtained from the pilot study, subsequent analyses were performed on PB samples taken at −3 days (pre-irradiation), 297 and 420 days post-irradiation, with only mice alive at the final blood sampling analysed for all time-points. A significant effect of time on the methylation of L1 (*P* = 0.047; ANOVA with repeated measures) and B1 (*P* = 0.044) elements was detected, indicating some fluctuations within individual mice and between mice over time (Figure 2). However, Bonferroni *post hoc* analysis indicated there were no significant differences in L1 or B1 methylation levels between the pre-irradiation samples and the final PB sample at 420 days post-irradiation (*P*>0.05). No significant effect on IAP element methylation was detected (*P* = 0.716, ANOVA with repeated measures), and for all three elements there was no effect of the 10 mGy irradiation (*P*>0.05).

### Sham and 10 mGy irradiated mice have reduced repeat element methylation levels compared to untreated, age-matched 17–19 month old mice in spleen and liver

Spleen and liver tissues were isolated from the sham and 10 mGy irradiated mice at the end of Study 2 (420 days) and examined for repeat element methylation changes. A comparison with spleen and liver tissues from a cohort of untreated age-matched mice was made. In spleen there were no significant differences in methylation levels between any of the groups for either the L1 elements (*P* = 0.925, ANOVA) (Figure 3A) or IAP elements (*P* = 0.182, Kruskal-Wallis) (Figure 3C). For the B1 elements, the sham and 10 mGy treated mice exhibited significantly lower methylation than the untreated mice (*P* = 2×10^−8^ sham vs. untreated; *P* = 1×10^−8^ 10 mGy vs. untreated, ANOVA with Bonferroni *post hoc*) (Figure 3B). In liver, there were no significant differences in methylation between any of the groups for either the L1 elements (*P* = 0.718, ANOVA) (Figure 3D) or IAP elements (*P* = 0.053, ANOVA) (Figure 3F). For the B1 elements, the sham and 10 mGy treated mice had significantly lower methylation than the untreated mice (*P* = 8×10^−6^ sham vs. untreated; *P* = 0.001 10 mGy vs. untreated, Welch ANOVA with Games-Howell) (Figure 3E). No significant differences between the 0 mGy and 10 mGy irradiated groups were identified for any of the repeat elements (L1, B1 or IAP) in either spleen or liver tissue.

### Pyrosequencing analysis demonstrates that sham and 10 mGy treated mice have lower B1 methylation levels than untreated mice in liver

A random selection of liver B1 element HRM amplicons from the untreated, sham and 10 mGy treated mice were pyrosequenced (*n* = 7 per treatment group). Analysis of individual CpG methylation levels (Figure 4A, Table S2) indicated that CpGs 1–3, 5 and 7 of the sham and 10 mGy treated mice were less methylated compared to the untreated mice with differences in methylation ranging from 1.8–4.0% (*P*<0.05, ANOVA with Bonferroni *post hoc*). There were no significant differences in the methylation levels between the sham and 10 mGy treated mice at any of the CpGs (*P*>0.05).

Examination of the mean methylation levels of the B1 elements (Figure 4B) demonstrated that both the sham and 10 mGy treated mice had a significantly lower NTS than the untreated mice (*P* = 0.0009, sham vs. untreated; *P* = 0.0004, 10 mGy vs. untreated; ANOVA with Bonferroni *post hoc*). There was no significant difference in the mean methylation of all CpGs pyrosequenced between sham and 10 mGy irradiated mice (*P*>0.05).

### Sham and 10 mGy irradiated mice have reduced liver global DNA methylation compared to untreated age-matched mice

The genomic methylation levels of a random selection of liver tissues from the sham and 10 mGy irradiated mice (Study 2) were analysed by LC-MS and compared to age-matched (17–19 month) untreated mice (*n* = 7–10 per treatment group) (Figure 4C). The sham and 10 mGy treated mice displayed lower relative mean methylation compared to the untreated mice however only the sham group reached statistical significance (*P* = 0.049, sham vs. untreated; *P* = 0.074, 10 mGy vs. untreated; Welch ANOVA with Games-Howell).

### L1, B1 and IAP repeat elements demonstrate tissue and repeat element dependent changes in DNA methylation in untreated mice of varying ages

Untreated mice from varying age groups (2, 4–6, 17–19 and 26 months of age; Table 2) were analysed by HRM in order to determine if age-related changes in repeat element DNA methylation could be detected. In spleen, mice exhibited increased methylation of L1 elements at 17–19 months of age compared to mice aged 2 months (*P* = 5×10^−5^; ANOVA with Bonferroni *post hoc*), 4–6 months (*P* = 0.045) and 26 months (*P* = 0.001) (Figure 5A). For the B1 elements, the 17–19 month old mice also exhibited increased methylation compared to mice aged 2 months (*P* = 1×10^−5^, Welch ANOVA with Games-Howell *post hoc*), 4–6 months (*P* = 0.0001) and 26 months (*P* = 0.008) (Figure 5B). There were no significant differences between any of the age groups for the spleen IAP elements (*P*>0.05) (Figure 5C) or liver L1 element methylation levels (*P*>0.05) (Figure 5D). When compared to the 2 month old mice, the liver B1 element methylation levels were lower in the 4–6 month (*P* = 0.006, Kruskal-Wallis with Mann Whitney and Bonferroni *post hoc*) and 26 month old mice (*P* = 0.005) (Figure 5E). The 26 month old mice also had significantly lower B1 methylation than the 17–19 month age group (*P* = 0.018). For the IAP elements, no significant differences between any of the age groups was detected with the exception of the 4–6 month old mice which displayed lower methylation levels relative to the 2 month age group (*P* = 0.002, Welch ANOVA with Games-Howell *post hoc*) (Figure 5F).

**Table 2:** Mouse numbers and age groups of untreated mice.

		*Male*			*Female*		
		*n*	Age (months)	*SD*	*n*	Age (months)	*SD*
**Age group (months)**	2	5	2	*0*	5	2	*0*
	4–6	4	4.5	*1*	5	5.4	*0.55*
	17–19	4	18.5	*0.58*	3	18.3	*0.58*
	26#				5	26	*0*

# Only female mice were available for this age group.

### Analysis of liver global DNA methylation levels in untreated mice of varying ages

LC-MS was used to analyse global DNA methylation levels in the liver tissues from untreated mice of varying age groups (2, 4–6, 17–19 and 26 months of age; Table 2). The 17–19 month old mice displayed higher relative methylation levels than the 2 month old mice, however the difference was not statistically significant (*P* = 0.08, Welch ANOVA with Games-Howell *post hoc*). There were no significant differences in methylation levels between any of the other age groups (Figure 6).

## Discussion

We sought to determine if a low dose of ionising radiation could modify the decline in DNA methylation that occurs with aging. Our study was based on two separate observations reported in a number of published studies: that aging mice and humans exhibit reduced DNA methylation which is proposed to contribute to the increased genomic instability associated with age; and that the radioadaptive response can induce a protective effect against DNA damage and markers of genomic instability [30], [33], [43], [59]–[64]. Human studies have shown a correlation between reduced DNA repeat element methylation and increased transposition and genomic instability [17], [19], [65]–[69], as well as an association between repeat element methylation levels and various aging-related diseases including a range of cancers [70], cardiovascular [71]–[73], neurological [74] and autoimmune disease [75].

Changes in repeat element methylation levels in the tissues examined in this study (spleen, liver and peripheral blood) have been documented and are associated with a range of cancers and autoimmune disorders [75]–[77]. Whether changes in repeat element methylation are the causative agent of these diseases is still unknown (reviewed in [70]).

Radiation studies indicate that high dose exposures can reduce global and repeat element methylation levels, which has also been correlated with increased transcription of these elements [78], [79]. However low dose radiation (<100 mGy) has been shown to induce protective mechanisms reducing aging-associated DNA damage [30], cancer incidence [31]–[36] and increasing lifespan [32], [37] in mice. Here we investigated whether a low dose of ionising radiation at a dose relevant to human diagnostic exposure could modulate DNA methylation levels and whether it could reduce or protect against aging related changes to DNA methylation.

Surprisingly, we did not detect an age-related decline in repeat element methylation levels in mouse PB. Although our hypothesis was based on previous studies demonstrating a decline in human PB methylation levels with age [6], [13]–[15], more recent studies in humans indicate that PB methylation levels may be influenced by the cell types present within the PB blood sample [80]–[82]. However, there are no studies reporting significant changes in white blood cell populations with aging in humans [83], and several mouse studies have reported no change in white blood cell populations in aging or following low dose irradiation [84]–[86]._ENREF_58_ENREF_58 Our study is the first to assess whether PB repeat element DNA methylation declines with age in mice and indicates that age has no detectable effect on L1, B1 or IAP repeat element methylation using the standard and sensitive methods employed here.

To determine if an age-related decline in repeat element DNA methylation occurs in other mouse tissues, a cross-sectional analysis of spleen and liver tissues from untreated mice of various age groups was conducted. Although we did not detect an overall loss in methylation at any of the repeat elements in the oldest mice, we did detect changes with age that were tissue and repeat element dependent. In spleen, middle-aged mice (17–19 months) exhibited increased methylation of L1 and B1 elements compared to all other age groups (Figure 5A, 5B) however no changes were evident in the IAP elements. The phenomenon of increased methylation at L1 repeat elements with age has been previously reported in humans, where CpG island-rich L1 regions exhibited increased methylation with age compared with CpG island-poor regions [16]. In our study, the increase in L1 and B1 methylation at 17–19 months was not evident in the 26 month old group however this may be due to the small sample size and the availability of only female mice in this age group (no male mice survived to 26 months). In liver, no consistent pattern of change was evident in the L1 or IAP repeat element methylation levels with the only significant difference identified in the 4–6 month old mice, which displayed reduced IAP methylation in comparison to the 2 month old mice. For the B1 elements in liver, the 4–6 month and 26 month old mice had significantly lower methylation levels than the 2 month old mice suggesting a possible age-related decline in methylation in this tissue. A reduction was not evident in the 17–19 month age group relative to the 2 month group and hence further investigations are required to determine whether a true age-related decline in B1 methylation occurs in liver. It has previously been shown in humans that there is a strong correlation between age-related changes in Alu element methylation levels (the human equivalent of B1 elements) and global methylation levels [13], [14], [46]. However the age-related changes observed in liver B1 element methylation levels were not reflected in the global methylation levels in our study. Interestingly, the pattern of change observed for the liver global methylation levels between the different age groups, although not significant, was similar to the pattern of age-related changes observed for the spleen L1 and B1 elements.

Although high dose radiation exposure has been shown to induce changes to both repeat element and global DNA methylation levels [57], [58], [87]–[93], the effect of a low dose has not previously been investigated. Here we investigated the effect of a low dose of ionising radiation on DNA methylation levels in PB longitudinally and the long term effect of low dose radiation in a radiosensitive tissue (spleen) and a tissue considered central to the aging process (liver). Despite using an assay sensitive to detecting small changes in DNA methylation levels, we did not observe any significant effect of a single whole body dose of 10 mGy X-rays on L1, B1 or IAP methylation in PB at any of the time-points examined over two separate studies. The spleen and liver tissues isolated from the mice at the end of the study did not demonstrate any significant effects of the 10 mGy irradiation administered at the start of the study. These results indicate that a low dose of ionising radiation, at a dose similar to human diagnostic exposures, does not induce detectable changes to murine repeat element DNA methylation in the tissues and at the time-points examined in this study.

While the 10 mGy X-radiation dose may have been too low to elicit any detectable changes to DNA methylation, we have previously demonstrated that mice irradiated with a single whole body dose of 10 mGy given at the same dose rate used here (13.9 mGy/min) induces a radioadaptive response causing significant changes in chromosomal inversion frequency in spleen and prostate [53], [54], [94]–[96]. A single dose of 10 mGy has also been shown to induce a radioadaptive response for a range of other endpoints [34], [36], [97]–[99]. Thus, although we know that a single whole body dose of 10 mGy can elicit a radioadaptive response in mice, our data suggests that a radioadaptive response for DNA methylation was not induced in the current study. While it is possible that any changes in methylation may have been below the detectable limit of the HRM assay used, this assay has previously been shown to be sensitive at reproducibly detecting small changes in repeat element DNA methylation, as small as 3% for the L1 elements [38]. In the present study, pyrosequencing analysis of the liver B1 elements indicated that a shift in NTS value of 1.0 between treatment groups is equivalent to a 2.1–2.8% change in methylation. Thus, if repeat element methylation was altered by the 10 mGy irradiation, we estimate any change to be less than this amount.

While DNA repeat elements are often examined in studies investigating the effect of aging and exposure to exogenous agents on DNA methylation, there is still no direct evidence indicating the amount of loss of methylation that is required to induce changes in genome-wide stability, unlike single gene loci where a change in methylation has been shown to alter expression, such as has been observed with the *MGMT* gene [100]–[102]. The reduction in liver B1 methylation levels in the sham and 10 mGy treated mice relative to the untreated group observed by HRM and pyrosequencing were also reflected in the liver genomic methylation levels where untreated mice had a mean methylation level of 6.65% and treated mice showed an 11–15% reduction in genomic methylation levels compared to the untreated group. Although this appears to be a considerable decrease, similar levels of change in global methylation levels have been reported in aging studies [103].

Interestingly, while no effect of the 10 mGy irradiation on repeat element methylation was detected in spleen or liver tissues taken at the end of the study, a comparison of the sham and 10 mGy irradiated tissues with age matched untreated mice indicated a significant reduction in B1 (spleen and liver) and IAP (liver) methylation levels in both treated groups of mice. This suggests that some aspect of the irradiation procedure may be responsible for inducing repeat element methylation changes in these tissues. While it may be argued that the observed changes are due to a mouse cohort phenomenon, the identification of changes in the B1 elements in spleen and liver with no changes detected for the L1 or IAP elements in either tissue suggests that this is not likely. The irradiation procedure involves transportation of the mice to a distant site and therefore a range of factors could be responsible for inducing this change including stress, exposure to environmental pathogens or a change in environmental conditions (e.g. temperature). Studies have previously demonstrated that stress and changes to environment can alter the epigenetic landscape including changes to repeat element methylation [104]–[107], however further investigations are required to determine if, and what aspect, of the irradiation procedure is responsible for the observed changes in the treated animals. Regardless of the cause, in the current study, the sham and 10 mGy treated groups responded to the irradiation procedure in a similar manner.

In summary, we present the first study to examine the temporal effect of a single dose of 10 mGy X-rays on repeat element methylation levels in mouse PB and the long term effect of this dose on repeat element methylation levels in a radiosensitive tissue (spleen) and a tissue considered central to the aging process (liver). Using an assay sensitive to detecting small changes in methylation, we did not observe any detectable effect of the 10 mGy irradiation on repeat element or global DNA methylation. This radiation dose is relevant to human diagnostic radiation exposures and suggests that a dose of 10 mGy X-rays, unlike high dose radiation, does not cause significant short or long term changes to repeat element or global DNA methylation. Furthermore, this study also demonstrates that, unlike human PB, mouse PB tissue does not display an age-related decline in repeat element methylation potentially indicating that mice may not be a suitable model system for analysing the effect of aging, for comparison with, or instead of, human studies.

## Supporting Information

Table S1 Primer sequences for amplification of repeat elements.
(DOCX)

Table S2 Pyrosequencing mean methylation of individual CpGs of liver B1 element HRM-PCR amplicons.
(DOCX)
