# Supplementary material for: A Single Whole-Body Low Dose X-Irradiation Does Not Affect L1, B1 and IAP Repeat Element DNA Methylation Longitudinally
Source: PLoS One. 2014 Mar 27;9(3):e93016. doi: 10.1371/journal.pone.0093016 (PMC3968115; doi:10.1371/journal.pone.0093016)
Supplement: Table S1 — Primer sequences for amplification of repeat elements. (DOCX) [file pone.0093016.s001.docx]

**Table S1: Primer sequences for amplification of repeat elements**

| Primer | Primer Sequence (5'-3') | Annealing Temperature (°C) |
| --- | --- | --- |
| F_unmeth_mLINE1 | G**T**T GAG G**T**A GTA **TTT** TGT GTG GG**T T** | 60 |
| R_unmeth_mLINE1 | TCC A**A**A A**A**C T**A**T CA**A** **A**TT CTC T**AA** C**A**C |  |
| F_unbiased_B1_Mm | AG**T** YGG G**Y**G TGG TGG | 52 |
| R_unbiased_B1_Mm | CTT T**A**T A**A**A CCA **AA**C T**AA** CCT C |  |
| F_unmeth_IAP_LTR | TTA TAT T**T**G T**T**G TTA TAA GAT GG **T** |  |
| R_unmeth_IAP_LTR | C**A**C CTA **AA**A C**A**T **A**TC ACT |  |

All primers are shown 5’- 3’.**Bold** typeface indicates bisulphite modified bases and biased CpG sites are underlined. A Y denotes a degenerate nucleotide – either a C or T.
