# Supplementary material for: A Single Whole-Body Low Dose X-Irradiation Does Not Affect L1, B1 and IAP Repeat Element DNA Methylation Longitudinally
Source: PLoS One. 2014 Mar 27;9(3):e93016. doi: 10.1371/journal.pone.0093016 (PMC3968115; doi:10.1371/journal.pone.0093016)
Supplement: Table S2 — Pyrosequencing mean methylation of individual CpGs of liver B1 element HRM-PCR amplicons. (DOCX) [file pone.0093016.s002.docx]

|  | ***Treatment group*** | | | | | | | |
| --- | --- | --- | --- | --- | --- | --- | --- | --- |
|  | *untreated* | | *0 mGy* | | | *10 mGy* | | |
| **CpG** | **Mean** | **SD** | **Mean** | **SD** | ***P*-value*** | **Mean** | **SD** | ***P*-value*** |
| **1** | 76.07 | 1.93 | 73.70 | 0.85 | *0.0259* | 73.56 | 1.60 | *0.0210* |
| **2** | 22.00 | 0.95 | 19.44 | 1.10 | *0.0028* | 19.17 | 1.51 | *0.0011* |
| **3** | 57.78 | 1.39 | 53.95 | 1.26 | *0.0010* | 53.45 | 1.32 | *0.00003* |
| **4** | 39.37 | 1.76 | 38.13 | 0.84 | *0.3040* | 37.88 | 1.28 | *0.1590* |
| **5** | 56.81 | 0.99 | 54.94 | 0.67 | *0.0004* | 55.00 | 0.41 | *0.0006* |
| **6** | 53.39 | 2.08 | 51.56 | 1.69 | *0.3370* | 51.09 | 2.33 | *1.0000* |
| **7** | 74.65 | 0.91 | 72.03 | 0.69 | *0.00001* | 72.30 | 0.64 | *0.00004* |

**Table S2: Pyrosequencing mean methylation of individual CpGs of liver B1 element HRM-PCR amplicons.**

*versus untreated group ; *P* <0.05, ANOVA with Bonferroni *post hoc*
